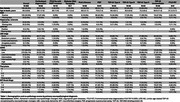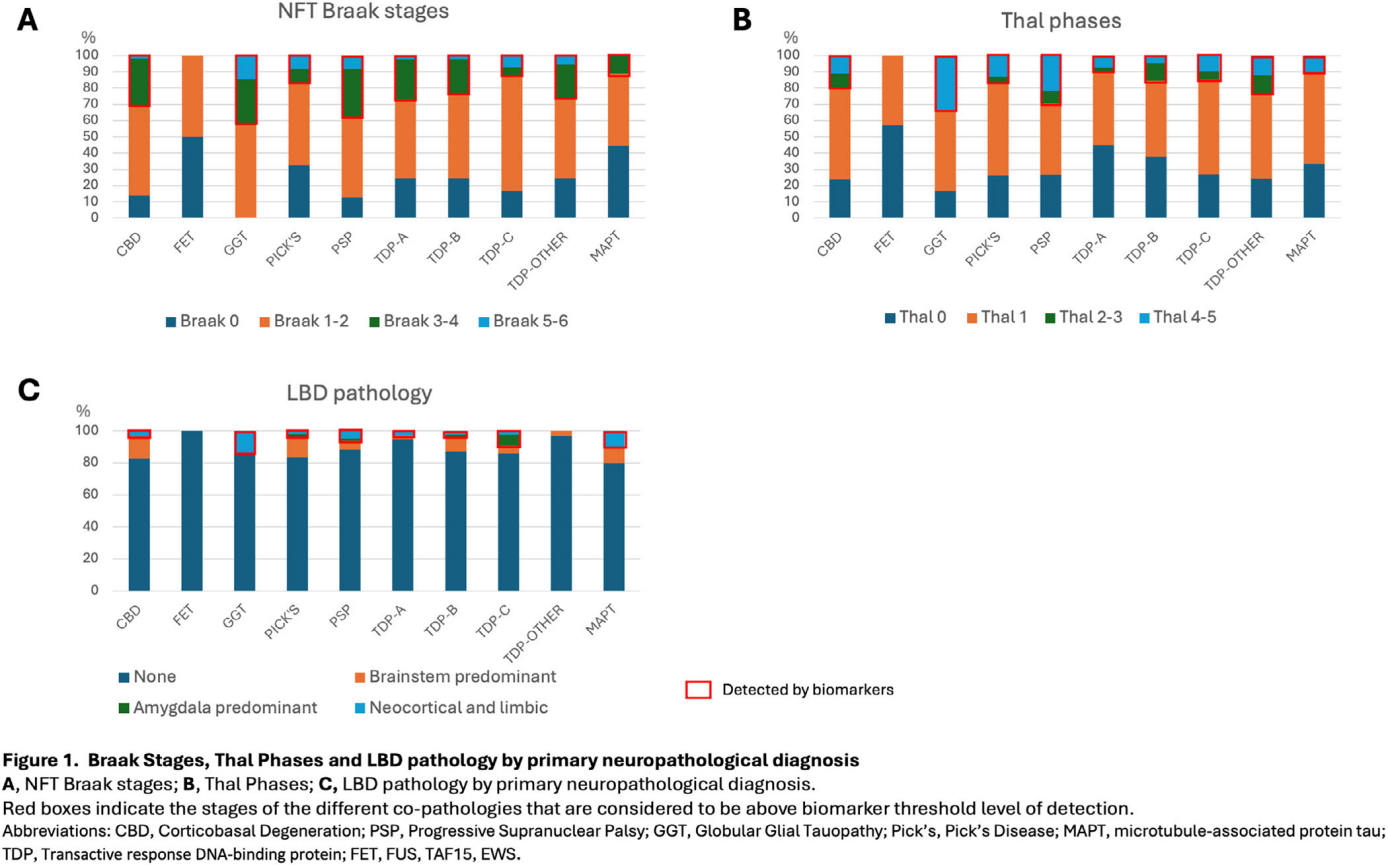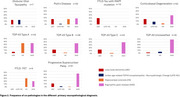# Neuropathological comorbidities in Frontotemporal Lobar Degeneration: Implications for Diagnosis

**DOI:** 10.1002/alz70855_107151

**Published:** 2025-12-24

**Authors:** Carolina Bidó Bello, D. Luke Fischer, Agathe Vrillon, Salvatore Spina, William W. Seeley, Howard J. Rosen, Maria Luisa Gorno Tempini, Isabel Elaine Allen, Bruce L. Miller, Lea T. Grinberg

**Affiliations:** ^1^ Global Brain Health Institute, San Francisco, CA, USA; ^2^ Memory and Aging Center, Weill Institute for Neurosciences, University of California, San Francisco, San Francisco, CA, USA; ^3^ Memory & Aging Center, Department of Neurology, University of California in San Francisco, San Francisco, CA, USA; ^4^ Department of Pathology, University of California, San Francisco, San Francisco, CA, USA; ^5^ Department of Neurology, Memory and Aging Center, University of California San Francisco, San Francisco, CA, USA; ^6^ Global Brain Health Institute, University of California, San Francisco, CA, USA; ^7^ Memory and Aging Center, Department of Neurology, University of California San Francisco, San Francisco, CA, USA; ^8^ Global Brain Health Institute, University of California, San Francisco, CA, USA, San Francisco, CA, USA, San Francisco, CA, USA; ^9^ Memory and Aging Center, UCSF Weill Institute forNeurosciences, University of California, San Francisco, San Francisco, CA, USA, San Francisco, CA, USA; ^10^ Department of Epidemiology and Biostatistics, University of California, San Francisco, San Francisco, CA, USA; ^11^ Global Brain Health Institute, University of California, San Francisco, San Francisco, CA, USA; ^12^ Memory and Aging Center, UCSF Weill Institute for Neurosciences, University of California, San Francisco, San Francisco, CA, USA; ^13^ Department of Pathology, University of California, San Francisco, California, USA., San Francisco, CA, USA

## Abstract

**Background:**

Frontotemporal lobar degeneration (FTLD) is an umbrella term encompassing neurodegenerative diseases that either feature tau, TDP‐43, or FET inclusions. As FTLD is associated with a younger age of onset, there has been less focus on studying age‐related co‐pathologies. The presence of co‐pathology affects clinical manifestations and has implications for diagnosis as well as management. To investigate the frequency and type of co‐pathologies in the FTLD spectrum, we examined a large *postmortem* clinicopathological cohort.

**Method:**

We included all FTLD cases at the Neurodegenerative Disease Brain Bank (NDBB) at UCSF collected between 2005 and 2024 (Table 1). All brains underwent standardized neuropathological assessment. We investigated the frequency of the most common age‐related neurodegenerative changes in primary neuropathological diseases including FTLD‐TDP43 type A (*n* = 43), type B (*n* = 48), type C (*n* = 43) and unclassifiable (*n* = 40); primary tauopathies: PSP (*n* = 91), Pick's disease (*n* = 50), CBD (*n* = 62), FTLD due to MAPT mutation (*n* = 10), GGT (all types, *n* = 7); and FTLD‐FET cases (*n* = 9).

**Result:**

In 403 cases (mean age at death: 69.2±9.6 years; 46.9% female), the most common co‐pathologies were AD neuropathologic changes (66.6%, all levels of ADNC considered, Figure 1) and argyrophilic grain disease (AGD, 33.5%). PSP and CBD had the highest percentages of intermediate ADNC levels. Hippocampal sclerosis (HS) was observed in 8.2% of the cohort (Figure 2). In FTLD‐TDP‐43, HS was more frequent in type A (34.9%) than in the other subtypes (2.1‐5%). HS was also observed in FTLD‐FET (33.3%) and Pick's (16%). Neocortical and limbic LBD co‐pathology at biomarker‐detectable levels was rare (3.0%).

**Conclusion:**

Our findings demonstrate that age‐related co‐pathologies are common in FTLD but at low burden, with AD neuropathologic changes and AGD being the most prevalent. The relative higher frequency of intermediate to advanced ADNC levels in PSP and CBD suggests that co‐existing AD pathology may contribute to the clinical presentation and progression of these disorders. HS, particularly in FTLD‐TDP43 type A, FTLD‐FET, and Pick's, highlights distinct vulnerabilities within the FTLD spectrum. Conversely, neocortical and limbic LBD co‐pathology was rare, reinforcing its limited role in FTLD. These findings emphasize the importance of comprehensive neuropathological assessments to refine diagnosis, improve prognostication, and guide future therapeutic strategies.